# Impairment of unconscious emotional processing after unilateral medial temporal structure resection

**DOI:** 10.1038/s41598-024-54868-2

**Published:** 2024-02-21

**Authors:** Wataru Sato, Naotaka Usui, Akihiko Kondo, Yasutaka Kubota, Motomi Toichi, Yushi Inoue

**Affiliations:** 1 Psychological Process Research Team, Guardian Robot Project, RIKEN, 2-2-2 Hikaridai, Seika-cho, Soraku-gun, Kyoto 619-0288 Japan; 2 National Epilepsy Center, Shizuoka Institute of Epilepsy and Neurological Disorders, Urushiyama 886, Shizuoka, 420-8688 Japan; 3 Health and Medical Services Center, Shiga University, 1-1-1 Baba, Hikone, Shiga 522-8522 Japan; 4 Graduate School of Medicine, Kyoto University, 53 Shogoin-Kawaharacho, Sakyo, Kyoto 606-8507 Japan

**Keywords:** Amygdala, Subliminal affective priming, Unconscious emotional processing, Unilateral temporal lobectomy, Visual half-field task, Amygdala, Consciousness

## Abstract

The role of the amygdala in unconscious emotional processing remains a topic of debate. Past lesion studies have indicated that amygdala damage leads to impaired electrodermal activity in response to subliminally presented emotional stimuli. However, electrodermal activity can reflect both emotional and nonemotional processes. To provide behavioral evidence highlighting the critical role of the amygdala in unconscious emotional processing, we examined patients (n = 16) who had undergone unilateral resection of medial temporal lobe structures, including the amygdala. We utilized the subliminal affective priming paradigm in conjunction with unilateral visual presentation. Fearful or happy dynamic facial expressions were presented in unilateral visual fields for 30 ms, serving as negative or positive primes. Subsequently, neutral target faces were displayed, and participants were tasked with rating the valence of these targets. Positive primes, compared to negative ones, enhanced valence ratings of the target to a greater extent when they stimulated the intact hemisphere (i.e., were presented in the contralateral visual field of the intact hemisphere) than when they stimulated the resected hemisphere (i.e., were presented in the contralateral visual field of the resected hemisphere). These results suggest that the amygdala is causally involved in unconscious emotional processing.

## Introduction

The role of the amygdala in unconscious emotional processing has long been a focus of research. Initial hypotheses on its involvement were based on evidence from animal studies^1,2^. Subsequent functional neuroimaging studies in humans demonstrated that the amygdala could be activated by subliminally presented emotional stimuli, such as emotional facial expressions^3–25^. Moreover, some research revealed that unconscious emotional processing can be routed through the subcortical visual pathway to the amygdala bypassing neocortical visual areas^5,11,16,25^. However, these findings are not consistent across all studies, and the extent of its role remains debated^26,27^.

Although functional neuroimaging studies provide correlational insights, lesion studies prove direct causal evidence of the functions of brain regions. Prior lesion studies have explored this matter in the amygdala by measuring electrodermal activity (EDA) in patients with amygdala damage^28,29^. For instance, one such study^28^ assessed patients with unilateral resection of anterior medial temporal lobe structures, including the amygdala. Subliminal and supraliminal presentations of negative and neutral scene photographs were made to their unilateral visual fields and EDA was recorded. Subliminally presented negative stimuli elicited a stronger EDA response when presented to the visual field contralateral to the intact hemisphere (i.e., stimulated the intact hemisphere) compared to the resected hemisphere (i.e., stimulated the resected hemisphere). Such findings suggest the role of the amygdala in unconscious emotional processing.

However, while EDA is valuable as a measure of emotional response, it has the limitation of potentially reflecting various cognitive processes and bodily responses^30^. For example, several psychophysiological studies have shown that EDA reflected memory processes^31^, which was reportedly associated with amygdala activity^32^. Other studies showed that EDA covaried with respiration^33^, which was suggested to be related to amygdala function^34^. Even when it signifies emotional reactions, it solely indicates the intensity of emotional arousal, either positive or negative, without providing valence information, which represents the qualitative spectrum from negative to positive^35^. A clue for this issue comes from previous lesion studies that have reported detrimental effects of amygdala damage on automatic processing of emotional stimuli, which does not necessarily reflect unconscious emotional processing^36–40^. For example, one study^39^ tested a patient with bilateral amygdala damage and healthy controls on a visual-search task that required participants to searched for an emotional (fearful or happy) or neutral facial expression among a crowd of neutral facial expressions. Although the controls detected facial expressions of fear and happiness more rapidly than neutral expressions, the patient did not. In another study^37^, a patient with unilateral amygdala damage had impaired reflexive eye movements toward briefly presented emotional facial expressions. Based on these lesion and neuroimaging data, we hypothesized that damage to the amygdala would compromise unconscious processing of emotional valence.

**Figure 1:**
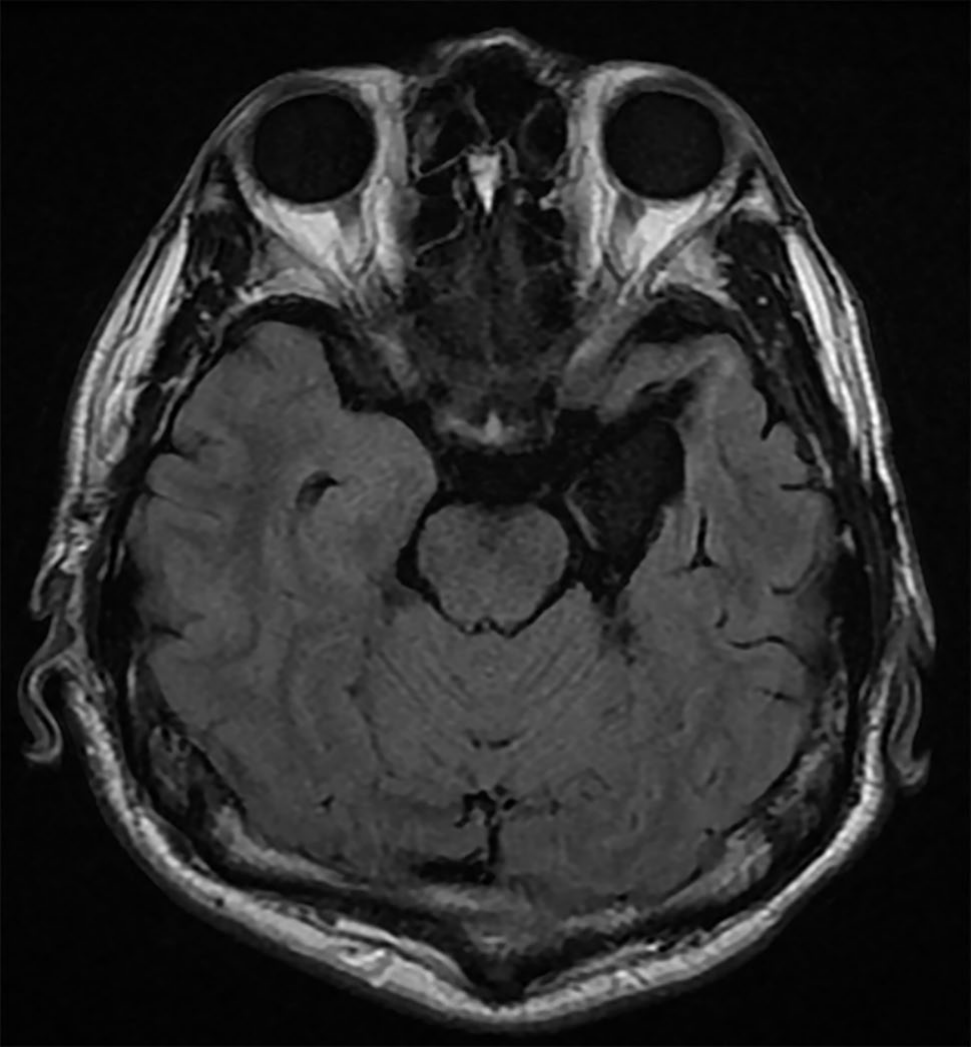
Representative anatomical magnetic resonance image of a patient after a medial temporal structure resection.

**Figure 2:**
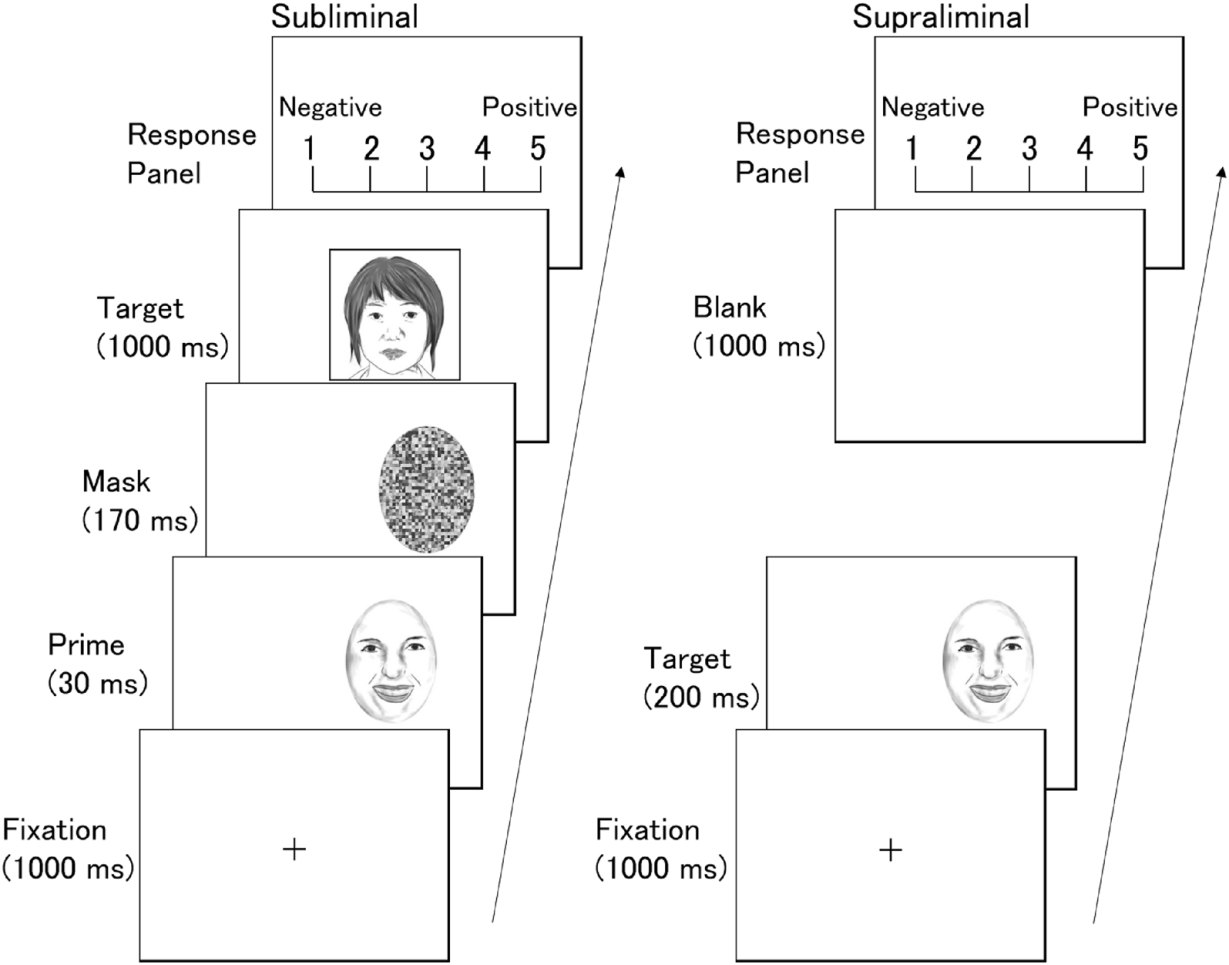
Illustrations of the trial sequence for the subliminal (left) and supraliminal (right) conditions. The prime facial expressions in the subliminal condition and target facial expressions in the supraliminal condition were presented dynamically. The stimuli were actual photographs of faces, while the images in the figure were produced by the authors.

To test this hypothesis, we evaluated 16 patients with unilateral temporal lobe resections, inclusive of the amygdala (Fig. 1 and Supplementary Fig. 1), utilizing a subliminal affective priming paradigm^41^ combined with unilateral visual presentation (Fig. 2). Dynamic fearful and happy facial expressions were presented briefly for 30 ms^42^ as negative and positive primes, followed by a mosaic mask, then neutral faces as target stimuli. Then the participants rated the emotional valence of these neutral target faces. Prior research suggests that in the affective priming paradigm, participants’ emotional assessment of the target is shifted toward the positive by unconscious positive primes, in contrast to negative primes^41,43^. This phenomenon is taken as evidence that emotions are unconsciously elicited and subsequently influence target evaluations^41^. This subliminal affective priming approach provides insights into the valence of unconscious emotion. We chose to present dynamic facial expressions because a previous study showed that dynamic expressions were more effective in inducing unconscious emotional responses than static expressions in a subliminal affective priming task^42^. We integrated this paradigm with unilateral visual field presentation. Given that visual stimuli presented to one visual field are predominantly processed in the opposing hemisphere^44^, we contrasted the valence ratings between the intact and resected hemisphere stimulation conditions as in previous studies^28,40,45^. We predicted that when primes stimulated the intact hemisphere (i.e., presented to the visual field opposite the intact hemisphere) compared to when they stimulated the resected hemisphere (i.e., presented to the visual field opposite the resected hemisphere), the distinction between positive and negative prime effects would diminish. To investigate conscious emotional processing, we also exploratorily presented dynamic facial expressions supraliminally (lasting 200 ms) and prompted participants to evaluate the valence of these expressions.

## Results

### Subliminal valence rating

**Figure 3:**
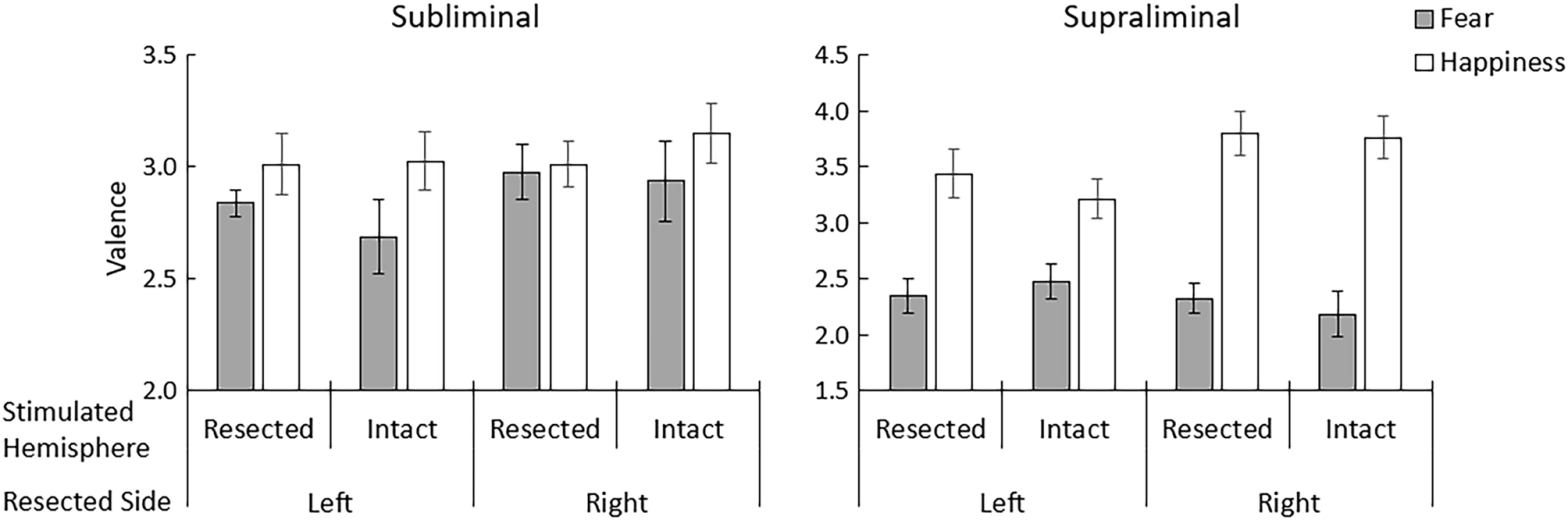
Mean ± standard error valence ratings for target neutral expressions following fearful and happy expression primes in the subliminal condition, and for fearful and happy expression targets in the supraliminal condition.

The valence ratings obtained under the subliminal and supraliminal conditions are shown in Fig. 3 and Supplementary Fig. 2. For the subliminal condition, valence ratings of target neutral faces were assessed following happy or fearful expression primes. The subliminal valence ratings were analyzed with a 2 (emotion: fear/happiness) × 2 (stimulated hemisphere: resected/intact) × 2 (resected side: left/right) repeated-measures analysis of variance (ANOVA) model. Our hypotheses of an interaction between emotion and stimulated hemisphere, and of a simple effect of emotion, were tested using planned contrasts (one-tailed). The effect of resected side, among other effects, were explored using two-tailed tests. The results revealed a significant interaction between emotion and stimulated hemisphere (*t*[14] = 1.78, *p* = 0.049, η^2^_p_ = 0.19), indicating that valence rating differences (happiness > fear) were greater when the intact hemisphere was stimulated than when the resected hemisphere was stimulated. Simple effect analyses revealed that the differences in valence ratings (happiness > fear) were significant when the intact hemisphere was stimulated (*t*[26.8] = 3.65, *p* < 0.001), but there was only a trend toward significant difference when the resected hemisphere was stimulated (*t*[26.8] = 1.41, *p* = 0.085). The three-way interaction was not significant (*t*[14] = 0.06, *p* = 0.948, η^2^_p_ = 0.00), suggesting that the interaction between emotion and stimulated hemisphere was not modulated by resection side. Besides, only the main effect of emotion (happiness > fear) was significant (*t*[14] = 3.26, *p* = 0.006, η^2^_p_ = 0.43); there were no other significant main or interaction effects (*t*[14] < 1.13, *p* > 0.282, η^2^_p_ < 0.09).

### Supraliminal valence rating

For the supraliminal condition, the same general linear modeling analyses with the above analyses were conducted. The interaction between emotion and stimulated hemisphere was not significant (*t*[14] = 0.43, *p* = 0.185, η^2^_p_ = 0.06). Besides, the main effect of emotion (happiness > fear) was significant (*t*[14] = 7.68, *p* < 0.001, η^2^_p_ = 0.81), and the interaction between emotion and resected side showed a trend toward significance (*t*[14] = 1.93, *p* = 0.074, η^2^_p_ = 0.21). There were no other significant main or interaction effects (*t*[14] < 1.68, *p* > 0.118, η^2^_p_ < 0.17).

### Forced choice recognition

To objectively measure the subliminal effects of the prime stimuli, a forced-choice recognition session was conducted following the valence rating sessions, consistent with earlier research^41,42,46,47^. Each trial followed the sequence used in the subliminal valence rating session. Subsequently, participants were presented with two photographs showing the same emotional expressions (fear or happiness), one of which had previously been shown as the prime in that trial; participants had to identify that face. The mean ± *SD* recognition accuracy was 46.9 ± 2.2%. One-sample *t*-tests confirmed that this recognition accuracy did not significantly differ from chance (*t*[15] = 1.40, *p* = 0.182, *d* = 0.35). These findings establish an objective criterion indicating the subliminal presentation of primes in this experimental setting^48^. Furthermore, debriefing interviews confirmed that participants did not subjectively detect the primes.

## Discussion

Our results highlight the more pronounced effect of positive versus negative primes when stimulating the intact hemispheres compared to the resected hemispheres. These findings are consistent with previous functional neuroimaging studies wherein the amygdala was activated in response to subliminally presented emotional stimuli^3–25^. In addition, these outcomes support findings from lesion studies that have suggested that damage to the amygdala impairs EDA activity in reaction to subliminally presented emotional stimuli^28,29^. However, previous studies have not established the causal role of the amygdala in unconscious emotional processing due to the correlational nature of neuroimaging evidence and the inherent limitations of EDA findings in determining specific emotional valence processing. Consistent with our results, several lesion studies showed that amygdala damage impaired automatic processing of emotional stimuli^36–40^, though these studies did not specifically describe impairment in unconscious emotional processing. To the best of our knowledge, this is the first study to demonstrate that the amygdala is crucial for facilitating unconscious emotional processing.

By contrast, results from the supraliminal condition did not show differences in whether the stimulated hemisphere was the resected side. We speculate that this difference reflects the distinct neural mechanisms guiding unconscious versus conscious emotional processing. Prior neuroimaging research has revealed differential visual pathways for processing subliminal versus supraliminal emotional stimuli; specifically, subcortical visual pathways are active during the former while both subcortical and cortical visual pathways are engaged during the latter^25^. Several neuroimaging studies consistently reported that conscious processing of dynamic facial expressions stimulates various cortical regions, inclusive of the superior temporal gyrus, fusiform gyrus, and inferior frontal gyrus, alongside the amygdala^49–51^. In conjunction with these data, our findings suggest that activation in neocortical regions may offset the impact of amygdala damage during conscious emotional processing.

Our results carry theoretical implications. First, they offer insights into the temporal dynamics of neural emotional processing involving the amygdala. The exact timing of amygdala engagement in emotion processing has been a subject of debate. Some researchers speculated that the amygdala performs emotional evaluations of stimuli at a more advanced stage, subsequent to the neocortical processing linked with the conscious perception of stimuli^26,27^. Conversely, others contend that the amygdala facilitates rapid, unconscious emotional processing^2,52,53^. Several neuroimaging studies that observed amygdala activation in response to subliminal emotional stimuli align with the latter perspective^3–25^. Similarly, intracranial electroencephalography studies have documented amygdala electrical activity in response to emotional stimuli emerging before 100 ms^54–57^. This precedes the neocortical activity linked with conscious perception, which is typically observed ~ 200 ms after stimulus onset^58,59^. Specifically, one of these studies^57^ found that the amygdala was activated in response to subliminally presented fearful versus neutral expressions, with an onset latency of 88 ms. Our findings provide lesion-based substantiation to these recording observations and underscore the early engagement of the amygdala in emotional processing.

Second, our results enrich understanding of the psychological interplay between emotion, consciousness, and cognition. The affect primacy hypothesis has previously been proposed, and suggests that stimuli undergo emotional evaluation prior to conscious cognitive processing^60^. This hypothesis, however, remains contested. Some researchers argue that cognitive processes in neocortical regions occur before the emotional processing in the amygdala due to the limited evidence supporting the early involvement of the latter in emotional processing, particularly in determining emotional valence^61,62^. Our research adds new evidence, demonstrating the crucial role of the amygdala in the unconscious processing of emotional valence, thereby reinforcing the validity of the affect primacy hypothesis.

Several limitations of this study should be acknowledged. First, the statistical power may have been inadequate to discern more nuanced effects, potentially obscuring evident functional hemispheric asymmetries. Previous functional neuroimaging studies have suggested that the right amygdala exhibits more pronounced activation than the left in reaction to swiftly presented emotional expressions^3,63,64^. Further studies with expanded samples would likely enhance our understanding of the neural mechanisms underlying unconscious emotional processing.

Second, while our subliminal rating task revealed that emotional behaviors could be triggered by unconscious causes, the task did not assess whether the emotional experiences were conscious or unconscious^65^. A previous lesion study has anecdotally reported that a cortical blindsight patient experienced feelings of familiarity in response to unseen photographs of his family^66^, suggesting that unconscious causes can trigger conscious experiences. In contrast, a study of healthy participants reported that subliminally presented emotional facial expressions modulated the consumption of fruit-flavored drinks but not the ratings for emotional experiences^67^. The data suggest that unconscious causes can elicit emotional behaviors but not conscious emotional experiences. Further studies are needed to investigate the subjective nature of the unconscious emotional responses associated with amygdala activity.

In conclusion, we examined a cohort of patients who had undergone resection of the unilateral temporal lobe structures, including the amygdala, utilizing the subliminal affective priming paradigm combined with unilateral visual presentation. The effect of subliminal emotional primes was more evident when stimulating the intact hemisphere as opposed to the resected one. These results indicate a causal role of the amygdala in unconscious emotional processing.

## Methods

### Participants

Sixteen patients (10 females, 6 males; mean ± *SD* age = 34.8 ± 12.4 years) with unilateral resection of medial temporal lobe structures due to pharmacologically intractable seizures participated in the study. We conducted a priori power analysis using G*Power 3.1.9.2 software^68^ to determine the necessary sample size, assuming that a paired *t*-test (one-tailed) would be used to compare the intact and resected hemisphere stimulation with an α level of 0.05 and a power of 0.80. Because the effect size was unclear, we assumed a medium-sized effect (*r* = 0.36). The results indicated a requirement of 12 participants. All participants had undergone surgery at least 1 year before the experiment. For most participants, seizures were effectively managed (*n* = 13, 2, and 1 for Engel classes^69^ I [free from disabling seizures], II [rare disabling seizures], and IV [no worthwhile improvement], respectively), and they were mentally stable during the experiment. The resection methods included selective amygdalohippocampectomy, involving the amygdala, anterior hippocampus, and parahippocampal gyrus in seven individuals; and anterior temporal lobectomy, involving the amygdala, anterior hippocampus, anterior lateral temporal neocortex (from the temporal pole to 4–5 cm), and parahippocampal gyrus in nine individuals. Postoperative magnetic resonance imaging validated the targeted resections in all patients (Fig. 1). Among the 16 participants, 8 (3 females, 5 males; mean ± *SD* age = 32.4 ± 13.4 years) and 8 (7 females, 1 male; mean ± *SD* age = 37.1 ± 11.7 years) had undergone resection of the left and right hemispheres, respectively. We assessed handedness using the Edinburgh Handedness Inventory^70^, confirming that all participants were right-handed. All had normal or corrected-to-normal vision and provided written informed consent after receiving a detailed explanation of the procedure. The Ethics Committee of Shizuoka Institute of Epilepsy and Neurological Disorders approved this study. The experiment was conducted in accordance with institutional ethical guidelines and the Declaration of Helsinki.

### Apparatus

We utilized a Windows-based computer (HP Z200 SFF; Hewlett-Packard Company, Tokyo, Japan) equipped with a 19-inch CRT monitor (HM903D-A; Iiyama, Tokyo, Japan) and operated using Presentation 14.9 software (Neurobehavioral Systems, San Francisco, CA, USA). The monitor had a resolution of 768 vertical × 1024 horizontal pixels and a refresh rate of 100 Hz, verified using a 1000 frames/s camera (EXILIM FH100; Casio, Tokyo, Japan). Rests for the chin and forehead ensured a consistent 0.57 m distance between the participant and the monitor.

### Stimuli

For the valence rating task under subliminal conditions, the prime stimuli consisted of grayscale photographs of 10 Caucasian faces (5 females and 5 males) selected from a standardized set^71^, depicting fearful, happy, and neutral expressions. Neutral expressions were utilized as a foundation for morphing animations and producing mosaic images. No participant recognized any of the displayed faces. The faces were displayed elliptically to eliminate peripheral cues such as hair. The visual angle for both the primes and mask was set at 7.0° vertical × 5.0° horizontal.

Using morphing software (FUTON System; ATR, Soraku-gun, Japan) on a Linux computer, dynamic facial expressions were generated from these photographs. Based on a neutral expression (0%) and an emotional expression (100%), two intermediate expressions with 34% and 66% intensities were produced. Images with 34%, 66%, and 100% intensity were displayed sequentially as dynamic clips; each image lasted for 10 ms, resulting in a clip duration of 30 ms. A neutral facial image was sectioned into a 50 × 40 grid and subsequently rearranged to create a mosaic image.

Grayscale photographs of 40 Japanese faces (20 females and 20 males) displaying emotionally neutral expressions were utilized as target stimuli. Initially, 65 facial images in a database of amateur Japanese models^72^ were presented to 14 healthy participants (not involved in the primary experiment). These individuals rated the stimuli on a 5-point scale. The 40 models rated as relatively neutral were chosen as target stimuli, randomly allocated to experimental conditions (emotion/stimulated hemisphere). These target stimuli measured 7.0° vertical × 7.0° horizontal.

For the valence rating task under supraliminal conditions, the images of facial expressions with 34%, 66%, and 100% intensity were presented sequentially for 60, 70, and 70 ms, respectively (total of 200 ms), as the target stimuli.

### Procedure

Participants underwent testing individually across four sessions: visual field assessment, subliminal valence rating, supraliminal valence rating, and forced-choice recognition. They were instructed to maintain focus on a central fixation cross (0.86° × 0.86°) displayed throughout the sessions.

#### Visual field assessment

The potential for visual field deficits among participants was assessed across four trials. Each trial began with a white fixation cross (0.86° × 0.86°) displayed at the monitor’s center for 500 ms. Subsequently, a target stimulus (a 1.0° white circle) was shown for 200 ms in the corner of a square region where a prime face would appear during a subliminal valence rating task. Participants were instructed to indicate the location of the target following the display of the fixation cross. None exhibited visual field deficits.

#### Subliminal valence rating task

Both the subliminal and supraliminal valence rating tasks included 40 trials, with 10 trials under each of the four experimental conditions: 2 emotions × 2 visual fields. All trials proceeded in a pseudorandomized sequence. Before the main tasks, participants completed five practice trials using Caucasian faces (primes) and Japanese faces (target stimuli) not used in the main experiment to acquaint themselves with the procedures of each task.

For each trial of the subliminal valence rating task (Fig. 2), a fixation cross was initially displayed at the center of the visual field for 1000 ms. Then a prime stimulus appeared for 30 ms in either the left or right visual field (with the inner edge approximately 5° from the center), immediately followed by a mask stimulus in the same position for 170 ms. The durations for both the prime and mask stimuli were established based on prior subliminal studies^42,73^ and a preliminary research outcome. Then a neutral face target was centered on the display for 1000 ms. Finally, the rating display was presented until participants completed their responses. Their task was to evaluate the valence of the target neutral face using a 5-point scale from “negative” to “positive.” Participants responded by pressing a designated key with the index finger of their right hand.

#### Supraliminal valence rating task

In each trial of the supraliminal valence rating task (Fig. 2), a central fixation point (a small cross) was presented for 1000 ms. Subsequently, the dynamic expression of the target was displayed for 200 ms, either in the left or right peripheral visual field, with the inner edge situated 5° from the center. This was followed by a blank screen for 1000 ms, and subsequently, the rating display was shown until a response was registered. Participants were instructed to rate the valence of the expression.

#### Forced-choice recognition task

For the forced-choice recognition task, five trials from each of the four (emotion × visual field) experimental conditions were selected at random, resulting in a total of 20 trials. Each trial followed the sequence used in the subliminal valence rating session. Subsequently, participants were presented with two emotional expression photos, one of which had been previously shown as the prime in that trial. Positioned in the upper and lower visual fields, the emotional states of these two facial stimuli were identical (either fear or happiness). Participants identified which face had been shown. This task assumed that participants, having developed visual awareness of the faces, could make choices based on rudimentary visual data.

### Data analysis

All statistical evaluations were performed using the SPSS 16.0J software (SPSS Japan, Tokyo, Japan). The valence ratings from both the subliminal and supraliminal tasks were analyzed separately because the task requirements were different between the conditions (i.e., the provision of ratings for static neutral expressions and dynamic fearful/happy expressions, respectively). The valence rating data were analyzed with a 2 (emotion: fear/happiness) × 2 (stimulated hemisphere: resected/intact) × 2 (resected side: left/right) repeated-measures ANOVA model. Given the initial research objectives, the interaction between emotion and stimulated hemisphere was assessed using a one-tailed test, while all other effects underwent two-tailed tests. The accuracy rate for the forced-choice recognition was analyzed with a one-sample *t*-test (two-tailed), compared against the chance level (i.e., 50%). Results were considered statistically significant at *p* < 0.05.

### Supplementary Information

Supplementary Information.

Supplementary Figures.

## Data Availability

The data supporting the findings of this study are available within the supplementary materials.
