# Supplementary material for: Impairment of unconscious emotional processing after unilateral medial temporal structure resection
Source: Sci Rep. 2024 Feb 21;14:4269. doi: 10.1038/s41598-024-54868-2 (PMC10881984; doi:10.1038/s41598-024-54868-2)
Supplement: Supplementary file 2 — Supplementary Figures. [file 41598_2024_54868_MOESM2_ESM.doc]

Impairment of unconscious emotional processing after unilateral medial temporal structure resection

Wataru Sato, Naotaka Usui, Akihiko Kondo, Yasutaka Kubota, Motomi Toichi, and Yushi Inoue

Supplementary data

Supplementary Figure 1. Anatomical magnetic resonance images of patients with unilateral temporal lobe resections. Left is shown on the right in the images.


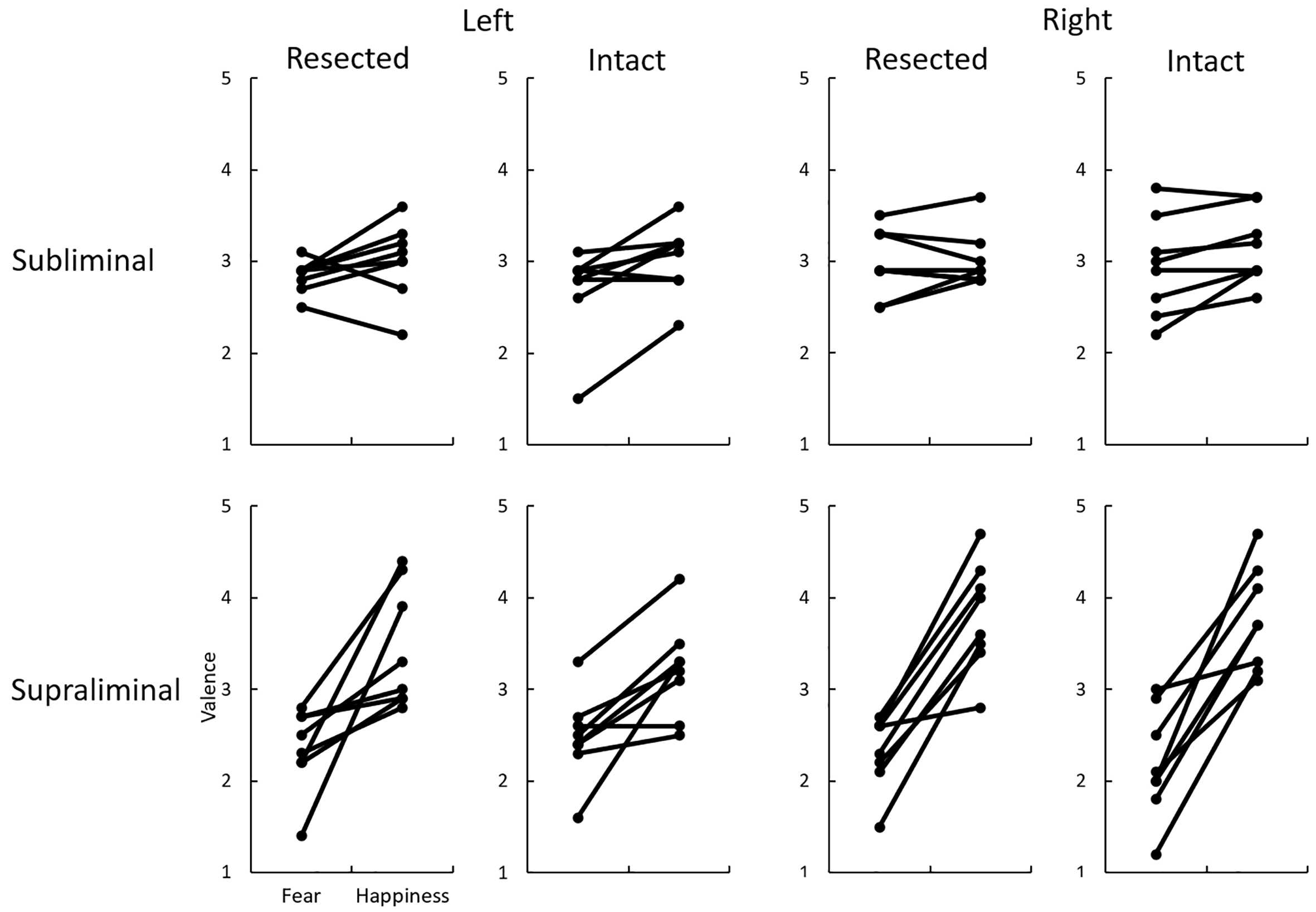


Supplementary Figure 2. Valence ratings for target neutral expressions following fearful and happy expression primes in the subliminal condition, and for fearful and happy expression targets in the supraliminal condition, for each participant. Left: Left hemisphere resected; Right: Right hemisphere resected; Resected: Resected hemisphere stimulation; Intact: Intact hemisphere stimulation.
